# Lutein and Brain Function

**DOI:** 10.3390/foods4040547

**Published:** 2015-10-09

**Authors:** John W. Erdman, Joshua W. Smith, Matthew J. Kuchan, Emily S. Mohn, Elizabeth J. Johnson, Stanislav S. Rubakhin, Lin Wang, Jonathan V. Sweedler, Martha Neuringer

**Affiliations:** 1 Division of Nutritional Sciences, University of Illinois, Urbana, IL 61801, USA; E-Mail: jwsmith5@illinois.edu; 2 Abbott Nutrition, Discovery Research, Columbus, OH 43219, USA; E-Mail: Matthew.Kuchan@abbott.com; 3 Jean Mayer US Department of Agriculture Human Nutrition Research Center on Aging, Tufts University, Boston, MA 02111, USA; E-Mails: Emily.Mohn@tufts.edu (E.M.S.); Elizabeth.johnson@tufts.edu (E.J.J.); 4 Departments of Chemistry and the Beckman Institute, University of Illinois, Urbana, IL 61801, USA; E-Mails: roubakhi@illinois.edu (S.S.R.); linwang@illinois.edu (L.W.); jsweedle@illinois.edu (J.V.S.); 5 Oregon National Primate Research Center, Oregon Health & Science University, Beaverton, OR 97006, USA; E-Mail: neuringe@ohsu.edu

**Keywords:** lutein, brain function, monkeys, animal models, carotenoids

## Abstract

Lutein is one of the most prevalent carotenoids in nature and in the human diet. Together with zeaxanthin, it is highly concentrated as macular pigment in the foveal retina of primates, attenuating blue light exposure, providing protection from photo-oxidation and enhancing visual performance. Recently, interest in lutein has expanded beyond the retina to its possible contributions to brain development and function. Only primates accumulate lutein within the brain, but little is known about its distribution or physiological role. Our team has begun to utilize the rhesus macaque (*Macaca mulatta*) model to study the uptake and bio-localization of lutein in the brain. Our overall goal has been to assess the association of lutein localization with brain function. In this review, we will first cover the evolution of the non-human primate model for lutein and brain studies, discuss prior association studies of lutein with retina and brain function, and review approaches that can be used to localize brain lutein. We also describe our approach to the biosynthesis of ^13^C-lutein, which will allow investigation of lutein flux, localization, metabolism and pharmacokinetics. Lastly, we describe potential future research opportunities.

## 1. Introduction

The carotenoid lutein is prevalent in nature, with important functions in the plant and animal kingdoms. It is an essential component of the chloroplast, playing a role in both light harvesting during photosynthesis and photo-protection when there is excess light. Lutein is found in a variety of human tissues and is especially concentrated in the macular region of the retina, where it is believed to protect against harmful blue light, oxidative damage and macular degeneration. Recently, it has become clear that lutein also preferentially accumulates in the human brain [1] and its content in neural tissue has been positively correlated with cognitive function [1,2,3,4].

Nonhuman primates are excellent animal models for brain physiology research. Only nonhuman primates share with humans the selective accumulation of lutein in both the retina and brain, thus overcoming the poor lutein absorption problems incurred with other animal models. Our team, composed of investigators from three Universities as well as Abbott Nutrition, has undertaken a project utilizing the rhesus macaque (*Macaca mulatta*) model. Our overall goal is to use this model to study the uptake and bio-localization of lutein in the brain and to assess the association of lutein content with brain function.

Several work streams are being carried out by our team that will be discussed in more detail in subsequent sections of this review. Prior work from two of the authors (E.J.J. and M.N.) studied lutein content of the primate eye and brain [5,6]. To expand this foundational research, lutein localization in various subcellular membranes in different anatomical regions of the macaque brain will be determined (E.J.J. and E.M.). Of particular interest is the specific accumulation and distribution of lutein and its relationships with areas associated with cognitive function. In addition, other researchers (J.V.S., L.W. and S.S.R.) are utilizing various mass spectrometry imaging techniques (e.g., matrix-assisted laser desorption/ionization-mass spectrometry imaging (MALDI-MSI) and laser desorption ionization-mass spectrometry imaging (LDI-MSI) [7,8] to assess the cellular localization of endogenous and stable isotope labeled lutein in brain regions, correlate its localization with other lipophilic compounds, and relate these neurochemical results to outcomes of morphological and functional tests. To enhance the team’s ability to localize and image lutein within brain compartments and membranes as well as to evaluate the pharmacokinetics of lutein in the brain, eye and other organs, other team members (J.W.S. and J.W.E.) are utilizing high lutein-producing carrot (*Daucus carota*) suspension cultures to biosynthesize ^13^C isotopically labeled lutein from ^13^C-glucose.

## 2. Development of the Monkey Model for Lutein Studies

**Figure 1:**
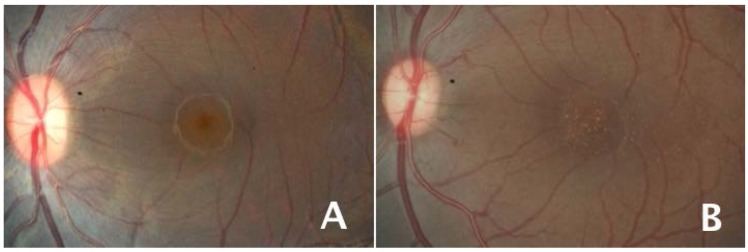
Visualization of macular pigment in two primate fovea. Retinal fundus photographs illustrating the presence of yellow macular pigment in the fovea of a normal rhesus monkey (**A**) and its absence in a monkey fed a diet devoid of carotenoids including lutein and zeaxanthin (**B**). The right image in (**B**) also illustrates numerous macular drusen in the monkey lacking macular pigment.

While it has been known since 2004 that lutein is found in the human brain [9], progress in testing lutein’s importance for brain function has been limited by the lack of appropriate animal models. Most commonly utilized animal models of nutrition and cognition have poor absorption and negligible tissue deposition of lutein. Only human and nonhuman primates have a macula with a central fovea, a structure that selectively accumulates lutein and zeaxanthin, resulting in the ophthalmoscopically visible yellow macular pigment (Figure 1). This highly selective and concentrated distribution may be mediated by localized expression of the StARD3 binding protein for lutein [10] and the GSTP1 binding protein for zeaxanthin and meso-zeaxanthin [11]. Similarly, but unlike common experimental animal models, only the brains of primates accumulate these compounds, with lutein being the predominant brain carotenoid in both infant and elderly humans and in macaque monkeys [1,2,12]. Recent studies provide evidence that the unique accumulation of xanthophylls in primate retinal and neural tissues is related to low binding and inactivity of the key carotenoid cleavage enzyme, BCO2, which in other species is effective in metabolizing these compounds [13]. Thus, only nonhuman primates can provide a relevant model for investigating the metabolism and function of xanthophylls in the brain.

## 3. Roles of Lutein and Zeaxanthin in the Retina as a Guide to Studies of the Brain

Evidence has accumulated for the past 30 years on the importance of lutein and zeaxanthin in retinal health [14,15], but only recently have investigations been extended more generally to explore the potential role of these nutrients in the nervous system. In the retina, lutein and zeaxanthin’s beneficial effects include the absorption and filtering of blue light and their role as powerful antioxidants. These actions are particularly critical because in this tissue the damaging effects of light exposure are combined with the retina’s high oxygen tension and potential for oxidative damage; the latter is increased by retinal photoreceptors’ high content of polyunsaturated lipids, particularly the long-chain omega-3 fatty acid, docosahexaenoic acid (DHA). Studies of the effects of lutein/zeaxanthin dietary deficiency and supplementation on the nonhuman primate retina provide evidence for protective effects of these compounds, as well as background for newer work on their role in brain function.

A series of studies on rhesus monkeys fed diets completely devoid of carotenoids from birth through the lifespan demonstrated a number of effects on retinal structure and health. First, the normal yellow macular pigmentation was absent (Figure 1B), as measured *in vivo* and biochemically [6,16,17]. Morphologically, there were changes in the foveal cell density of the retinal pigment epithelium (RPE), the cell monolayer directly behind the retina that is in close contact with the photoreceptors and is critical for their nutrient supply and processing of waste products. Whereas monkeys fed a normal diet showed a peak in RPE cell density in the fovea, the xanthophyll-free monkeys had a dip at the center of the fovea [17], possibly due to dropout of RPE cells. Signs of age-related macular disease were also accelerated. Drusen are deposits under the retinal pigment epithelium that are the hallmark of early to intermediate age-related macular degeneration; they occur spontaneously in aging macaque monkeys. Many of the xanthophyll-free animals developed drusen at unusually early ages, suggesting acceleration of processes involved in aging and macular disease. Finally, when the foveal and perifoveal areas of their retinas were exposed to small spots of intense coherent blue light, the xanthophyll-free group’s foveal area showed significantly larger areas of damage than normal animals’ foveal area, where macular pigment density is high [18]. In contrast, the perifovea, outside the region of normally high macular pigment, showed no difference in degree of damage between the groups, confirming that the difference in foveal damage was due to protection by the macular pigment. Supplementation with pure lutein or pure zeaxanthin resulted in increases in blood and tissue levels of the corresponding carotenoid, the appearance of macular pigment, and lessening of foveal blue light damage to near the level of normal diet control animals [6,18,19].

In humans, lutein/zeaxanthin intake and blood levels have been associated in some, but not all, studies with a reduced risk of age-related macular degeneration (AMD), particularly its more advanced forms (reviewed in [20]). AMD is the most prevalent cause of vision loss in the elderly. Its pathogenesis is believed to involve oxidative damage, inflammation and activation of the immune system, particularly the complement pathway, all of which are reported to be ameliorated by xanthophylls [21]. In a large randomized, double-blind clinical trial by the National Eye Institute, supplementation with lutein and zeaxanthin, combined with the antioxidant combination of vitamins C and E plus zinc with copper, was found to reduce the progression of AMD to its advanced forms by 10% in comparison with groups receiving the antioxidant mixture only [22].

## 4. What is Known about Lutein and Cognition/Brain Function in Humans?

As stated above, lutein and its isomer zeaxanthin are the only carotenoids that are selectively deposited in the fovea to form the macular pigment. Given that, like the brain, the retina is composed of neural tissue, lutein is also being evaluated for a role in cognitive function [1,2,12]. Furthermore, cognitive impairment has been shown to be related to age-related eye diseases [23], suggesting that similar risk factors are involved in their etiologies.

As in the retina, lutein is the dominant carotenoid throughout human brain tissue, including regions controlling various aspects of cognition [12]. Given that similar mechanisms are likely to control the selective uptake of lutein and zeaxanthin in both the retina and brain, it is not surprising that macular pigment density was found to be significantly correlated with their levels in matched brain tissue [5,24]. Therefore, macular pigment density appears to be a useful biomarker of lutein concentrations in brain tissue. This relationship may explain the significant correlation found between macular pigment density and cognitive function in healthy adults [2,3,4]. Macular pigment density also has been found to be significantly related to multiple measures of temporal processing speed [25,26], an important aspect of sensory and cognitive function. Furthermore, examination of a relationship between cognition and lutein levels in brain tissue of adult decedents from a population-based study found that among the carotenoids, only lutein was consistently associated with a wide range of cognitive measures including executive function, language, learning and memory [1]. These associations remained statistically significant after controlling for potential confounding factors.

Although evidence is accumulating on a role for lutein in cognitive function, the studies discussed above are correlative, and thus do not establish a causal relationship. However, a four-month double-blinded, placebo controlled trial in older women revealed that those who were supplemented with lutein (12 mg/d), DHA (800 mg/d), or a combination had improved verbal fluency scores compared to a placebo group. Memory scores and rate of learning improved significantly in the combination treatment group, which also trended toward more efficient learning [27].

## 5. Proposed Mechanisms of Lutein Action

Based on the observations reviewed above, research evaluating the mechanisms by which lutein influences brain function is warranted. With the exception of protection from blue light, most of the mechanisms by which xanthophylls appear to protect the retina from damage may apply to the brain as well. In addition, there may be other roles of xanthophylls in both the retina and brain that are not yet fully understood. Examining the regional and subcellular membrane distribution of lutein and zeaxanthin in the brain will provide a critical starting point for understanding these novel functions.

Lutein is likely neuroprotective through its role as an antioxidant, an effect that may apply globally and not be limited to specific regions or cognitive domains. The brain is especially vulnerable to free radical attack due to its high polyunsaturated fatty acid content and high metabolic activity. Lutein is differentially localized to membrane domains rich in polyunsaturated fatty acids including DHA, and therefore is well positioned to block oxidation of these vulnerable lipids [28]. Inhibition of DHA oxidation not only helps to maintain membrane structure and fluidity but also preserves DHA so it remains available for cleavage and conversion into anti-inflammatory molecules [29]. Although the mechanisms by which lutein and DHA may function together is unclear, elevated DHA oxidation has been observed in the brains of Alzheimer’s disease and cognitively impaired patients; [29], therefore, this potential lipid-protective action by lutein may, in part, explain the relationship between lutein and cognition. However, lutein may function through several other independent mechanisms to affect brain function. Lutein has been suggested to modulate functional properties of photoreceptor, synaptic and other membranes along with changes in their physiochemical and structural features [28,30]. Unlike nonpolar carotenoids, lutein and zeaxanthin have polar groups at each end of the molecule that give them a membrane-spanning configuration in membrane lipid bilayers, where they are believed to assume a perpendicular or nearly perpendicular orientation. Together with their high solubility in membranes, this characteristic can strongly influence membrane properties including fluidity, ion exchange, oxygen diffusion and membrane stability [28] and may influence interneuronal communication through effects on gap junctions [31]. Studies evaluating the relationship between lutein concentrations in neural membranes and biomarkers of cell health and viability will provide insights to the mechanisms by which lutein functions in cognition.

Cognitive impairment can be caused by brain cell damage, dysfunction, and death, and in particular by loss of neural connectivity. Given that brain viability is affected by the structural integrity and function of its membranes, lutein located within these brain membranes may influence cognition by maintaining cell viability through inhibition of these processes. Lutein is known to accumulate in cell membranes [32] and axonal projections [33], due to its amphipathic structure [30]. Membranes vary in composition among different brain regions, including those controlling cognition [34,35]. The prefrontal cortex, striatum, and hippocampus all differ in lipid composition [35,36], and each have a role in different cognitive functions, although they interact with each other through complex circuits [37,38]. The prefrontal cortex controls higher-order cognitive functions including planning and decision-making, problem solving, abstract rule learning, cognitive flexibility and spatial working memory. The striatum, in addition to its role in control of motivated movement, is also involved in working memory, abstract rule learning, and attention control. The hippocampus is critical for the formation and consolidation of declarative or explicit memories.

Membrane composition also varies among cell types and cellular compartments [39], including mitochondrial, nuclear, myelin, and neuronal plasma membranes (PM). These membranes each have unique functions, many of which determine cell viability. Therefore, lutein accumulation in specific brain regions and membrane types may provide clues to its function.

For example, the neuronal PM participates in cell survival signaling pathways [40]. PM composition and fluidity can modulate the accessibility of membrane receptors to other molecules, or to the membrane itself [40]. As a result, these factors dictate membrane receptor and transporter aggregation and protein expression at the cell surface, thereby leading to changes in cell signaling that determine cell viability [40]. Thus, lutein accumulation in neuronal PMs may influence cell survival signal transduction.

Mitochondrial health is also an important determinant of overall cell viability. In healthy cells, reactive oxygen species (ROS) produced by mitochondria as a byproduct of ATP production are neutralized by the antioxidant defense network. However, an imbalance between these processes, due to either increased ROS production or decreased antioxidant capacity, leads to oxidative stress and cell damage [41]. Damaged mitochondria have elevated ROS production due to less efficient ATP synthesis, and can cause cell death [41]. Therefore, lutein accumulated in the mitochondria may protect this organelle from damage.

In the nucleus, gene regulation and DNA damage affect cell viability and are related to aging and cognition [42], and these processes may be altered by the presence of lutein. Myelin, which forms an insulating sheath around axons to increase nerve impulse speed, is vital for efficient neuronal communication. Myelin abnormalities are linked with a variety of neurological problems including impaired thinking and memory [43]. Therefore, accumulation of lutein in myelin may be indicative of a role in influencing structural integrity of myelin and maintaining proper communication between neurons.

## 6. Approaches for Evaluation of Lutein in Micro-Dissected Brains

**Figure 2:**
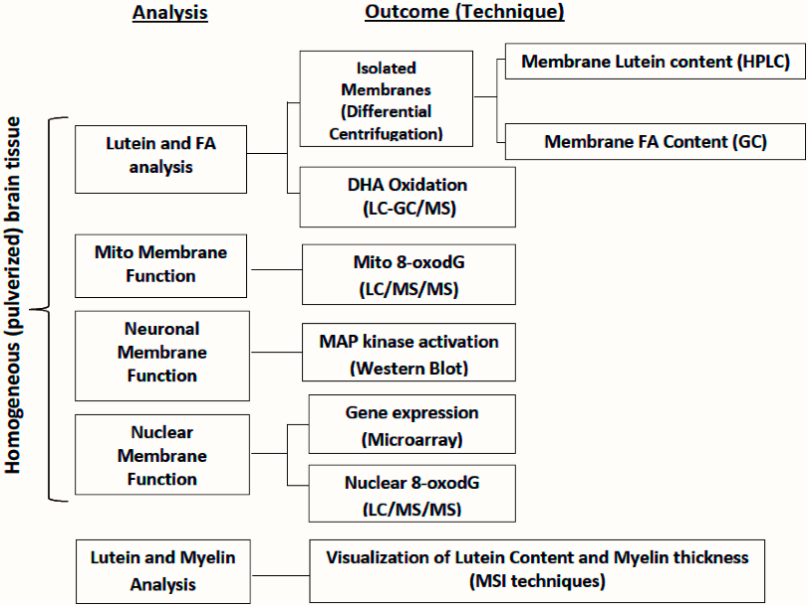
Approaches for assessing the association between lutein and brain function.

Measuring lutein concentration in neuronal, myelin, nuclear, and mitochondrial membranes from different regions of the primate brain is a key initial step in determining mechanisms by which lutein might influence cognitive function. To accomplish this, we outline one approach for its measurement. First, homogenized brain tissue is subjected to sequential centrifugation steps to first isolate the nuclear membrane as well as the crude membrane pellet containing myelin, neuronal, and mitochondrial membranes (Figure 2) [39]. The crude membrane pellet is then applied to a Ficoll density gradient and centrifuged for isolation and separation of myelin, neuronal, and mitochondrial membranes. All membranes are then purified via centrifugation. For lutein analysis, homogenized membranes are saponified and carotenoids are extracted with hexane and quantified by a reverse phase high performance liquid chromatography (HPLC) system and photodiode array detector [1].

For the assessment of the relationship between membrane lutein and DHA, fatty acid profiles can also be determined in isolated membranes to investigate whether lutein and DHA accumulate together. In the analysis of fatty acids, membrane lipids are extracted using the Folch method, saponified, methylated, and analyzed using gas chromatography [44]. To assess lutein’s role as an antioxidant in neural tissue, products of DHA oxidation (neuroprostanes, NP) are measured in the brain, and concentrations can be related to those of lutein. Lipids from homogenized brain tissue are extracted using the Folch method and saponified [45]. NP are extracted with ethyl acetate, derivatized to pentafluorobenzyl (PFB) esters, and isolated by HPLC using an amino column with a hexane/2-propanol gradient [46]. Collected NP fractions are converted to trimethylsilyl ether derivatives and quantified using GC-MS-NCI-SIM [45,46].

Since each neural membrane has a specific function that influences cell viability, the lutein concentration in a particular membrane and its relationship to markers of cell health and viability may provide insight into its role in brain function (Figure 2). Lutein’s roles in regulation of cell survival signaling, as well as mitochondrial and nuclear health and function, can be investigated by a variety of methods including the following: activation of cell survival signaling (e.g., MAP kinase) can be measured by Western blots, DNA damage by LC/MS/MS to quantify 8-oxo-7,8-dihydro-2′-deoxyguanosine in mitochondrial and nuclear DNA [47], and gene expression in brain tissue by RNA sequencing. In each case these measures can be related to lutein content in a specific membrane from the same brain sample. Lutein’s role in maintaining myelin structural integrity can be investigated using MSI techniques to visualize both myelin lutein content and myelin thickness.

## 7. Approaches for Visualization of Lutein Localization in Monkey Macula/Brain

Macular pigment [48] contains lutein, zeaxanthin, and meso-zeaxanthin; the detection and distribution of these compounds in the eye can be determined optically [49,50,51], and via spatially defined sampling and analyses by liquid chromatography with several detection techniques [52,53]. The optical methods (reviewed in [54]) include heterochromatic flicker photometry (a psychophysical approach [51]), two wavelength reflectometry [19], resonance Raman spectroscopy ([55], reviewed in [49]), fundus reflectance [56], and autofluorescence imaging [50] including two wavelength fundus autofluorescence [57]. Most optical methods use the property of carotenoids to absorb light at 460 nm and their low absorption in the spectral region above 500 nm. These methods benefit from the specific differential distribution of carotenoids in the eye with high levels in macula of retina. Therefore, the peripheral retina can act as a negative control/reference in the same experiment. Although optical methods can be used *in vivo* [49] and often produce quantitative information, the weakness of these methods is that they do not distinguish among the different carotenoids.

**Figure 3:**
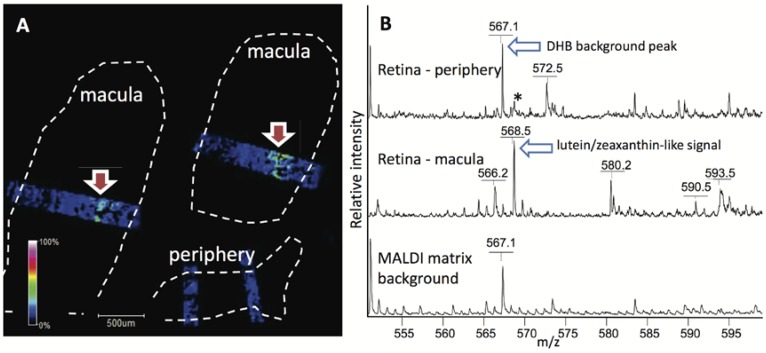
Lutein/zeaxanthin-like signal distributions in 10 μm thick sections of monkey retina detected using MSI. (**A**) Distribution of lutein/zeaxanthin-like signal in macula *vs*. periphery of retina. Only small rectangular areas in each retina section were examined (blue color). White and red colors represent highest relative signal intensities. Dashed line outlines areas occupied by tissue sections. (**B**) Representative mass spectra acquired from the examined retina regions. Asterisk marks likely matrix-related signal contributing to low intensity ubiquitous background.

Mass spectrometry imaging (MSI) is an information-rich tool for multiplexed characterization and localization of metabolites in tissues and organs including the eye and brain. MSI has been used to characterize distributions of a variety of lipid species in different areas of the eye [58,59,60,61,62,63,64,65]. Human [60,64], salamander [59], rabbit [58], porcine [61,62,63], and mouse [62,65] eyes have been investigated and images obtained of lipid distributions with up to 8–10 μm spatial resolution [59,65] and ±0.005 Da mass accuracy [61]. Our goal is to localize the carotenoids in the eye and brain using MSI. Details of our experimental approaches were previously published [66,67,68]. The well-defined localization and relatively high concentration of lutein in the macula makes it an effective model for the method optimization needed to observe carotenoids in the brain. We have used the monkey retina to develop methods for lutein detection in tissues that were fresh frozen and processed for MSI. Our results demonstrate the presence of signal characteristic of the macular pigments lutein, zeaxanthin and/or meso-zeaxanthin in the macular region but not in the peripheral retina (Figure 3). In these preliminary experiments, MALDI-MSI spatial resolution is sufficient to localize the lutein/zeaxanthin/meso-zeaxanthin signal to the ~100 μm thick Henle fiber layer of the monkey macula. Unfortunately, individual determination of these three isomers is problematic when performing MS imaging and localization measurements, as they cannot be distinguished based on molecular mass alone. ^13^C-lutein has a distinct molecular mass and monoisotopic distribution from endogenous carotenoids. Therefore, we plan to determine the spatial localization and concentration of lutein in eye and brain by applying MSI to samples from macaques given ^13^C-lutein. Importantly, specific spatial localization of lutein should aid our understanding of its function. The three carotenoids have differential distributions in macula, as previously found by biochemical methods for both human [52] and nonhuman primates [69]. Zeaxanthin is preferentially localized in the central macula region, lutein mostly is present in peripheral macula, and meso-zeaxanthin is concentrated in the central fovea.

## 8. Approaches for Biosynthesis of ^13^C-Lutein

Several challenges face scientists striving to elucidate carotenoids’ *in vivo* metabolism and mechanisms of bioactivity. As some carotenoid metabolites are not only found in foods but also can be produced by metabolic cleavage of the parent carotenoid *in vivo* (e.g., the β-carotene metabolite β-apo-10′-carotenal), there exists the problem of distinguishing endogenous pools of an analyte from those ingested, especially at specific time points. Second, carotenoids and/or their metabolites may be present in various tissues of the body in exceedingly low concentrations, testing the limits of photometric or MS detection after chromatographic separation. Isotopically labeled carotenoid tracers may be used to overcome both of these challenges. The study of β-carotene and vitamin A has benefitted greatly from the availability of such tracers [70,71] and if similar progress is to be made in the study of lutein, isotopically labeled lutein is needed more readily, in greater quantities, and at reasonable cost.

Methods of production of carotenoid tracers, including lutein, can be characterized by two general strategies: total chemical synthesis and isolation from biofactories. Total chemical synthesis of lutein has been previously described, [72] and isotopic labeling by this approach is therefore possible. However, synthesis of the masses of labeled tracer needed for feeding studies is cost-prohibitive for most investigations. Additionally, production of uniformly labeled or highly enriched carotenoids is both technically challenging and again, highly expensive.

Biofactories, in turn, are engineered systems centered on co-opting the metabolism within biological organisms to produce compounds of interest. Organisms used as biofactories may be microbial (bacteria, fungal, and yeast) or botanical. While much work has been done on the production of carotenoids and carotenoid tracers in microbial biofactories [73], some of it by our own group [74], this review will focus on botanical methods.

Most efforts to isotopically label carotenoids in biofactories have done so by utilizing carbon isotopes (sTable 1^3^C or radioactive ^14^C), rather than hydrogen isotopes (sTable 2H or radioactive ^3^H). In botanical systems, atmospheric carbon dioxide enters into the Calvin cycle and through several steps, is fixed into sugars. These carbohydrates serve as substrates for mitochondrial respiration and the synthesis of downstream metabolites, including carotenoids. Consequently, introduction of isotopically labeled carbon to botanical biofactories for incorporation into carotenoids can be accomplished by growing plants or cultured cells in the presence of labeled CO_2_ or sugars (sucrose or glucose). Especially of concern when working with radioactive ^14^C, containment of labeled sugars is much easier than labeled CO_2_. As a result, carbohydrates have been the source of labeled carbon in our work [75,76], as well as that of others. Additionally, differences in photosynthetic mechanisms between plant species have been shown to impact the distribution of carbon isotopes in downstream metabolites. C_3_ photosynthetic plants fix carbon dioxide into the three-carbon molecule 3-phosphoglycerate during their initial step of photosynthesis, while C_4_ plants sequester carbon in four-carbon oxaloacetate [77]. The enzymes which catalyze these initial carboxylation reactions differentially discriminate against atmospheric ^13^CO_2_, such that C_4_ photosynthetic plants such as corn incorporate ^13^C label from CO_2_ into lutein more effectively than C_3_ plants such as marigold and carrot [78]. However, this limitation is not encountered when supplying carbon label in the form of labeled sugars, therefore bypassing carbon fixation and photosynthesis.

Optimization of botanical biofactory systems requires attention to both total tracer mass yield, as well as isotopic labeling efficiency. Production of a low-enrichment tracer is less ideal than a uniformly labeled tracer, since each subsequent atomic label provides additional mass resolution from the native-mass carotenoid. In our previous work using a tomato cell suspension culture system to produce labeled lycopene and phytoene [75,76,79,80], we optimized cell line selection, cell culture media, culture conditions, culture duration, bleaching herbicide treatments, and carotenoid extraction methods to isolate maximal masses of carotenoid tracer. Use of a repeated batch culture system increased the efficiency with which harvested lycopene was uniformly labeled with ^13^C. In this system, tomato cells were grown in media containing ^13^C-glucose for three serial 9-day-long “loading” cycles, with each cycle providing inoculum for the subsequent loading cycle. With this method, the cellular ^13^C carbon pool is enriched with each loading cycle and in the final 18-day-long “labeling” cycle, the accumulated ^13^C is efficiently incorporated into lycopene. Compared to the natural ^13^C abundance of 1.1%, this method resulted in 93% ^13^C isotopic purity in the harvested lycopene tracer [76]. We are currently using similar approaches to maximize lutein yield and labeling efficiency from a carrot cell suspension culture system, with uniformly labeled ^13^C-glucose again as the source of labeled carbon. After screening 64 different cell lines, we have selected a target cell line, derived from the root tissue of the *Amarillo* carrot varietal, in which to conduct our lutein production and labeling. Experimentation determined that lutein yield in our selected cell line was increased by light culture conditions (*vs*. dark), higher light intensity (*vs*. lower), and Gamborg B5 cell culture media (*vs*. Murashige and Skoog). In contrast, there was no difference in lutein yield when glucose was provided as a sugar source in place of sucrose. Additionally, we have been able to optimize extraction methods for isolation of lutein, with recovery of >97%. Further optimization may investigate the effects of varying light source wavelength or application of growth inducers and carotenogenic stimulants in order to increase yields above current levels of 4.0 μg lutein/g wet cells.

In summary, the availability of sufficient amounts of affordable, high-purity (isotopically and chemically) lutein tracer will enable much-needed investigation into the biodistribution, pharmacokinetics, metabolism, and biological functions of this abundant dietary carotenoid.

## 9. Conclusions and Future Research

Accumulation of lutein and zeaxathin in the eye and brain only occurs in higher primates and humans. The high spatially-dependent concentrations found in the retina of these species led to research resulting in wide acceptance of their importance for retinal health across the life span. More recently, cognitive status has been correlated with ocular disease risk factors, macular pigment density and lutein intake. More direct evidence has been provided by positive correlations between pre-mortem cognitive function and post-mortem brain lutein concentrations [1], and by a controlled supplementation randomized clinical trial [26]. Macular pigment density also has been positively associated with temporal processing speed [25,26]. These findings suggest that lutein may directly impact brain function in addition to its effects on the retina, and they have intensified interest in identifying functions of lutein in the brain.

Novel mechanisms of action of dietary components are typically first understood in animal studies and then corroborated in clinical trials. While blood, secretions and excretions can be routinely sampled and analyzed in humans, target organs such as brain and retina cannot. Animal models are therefore especially useful assuming that absorption, transport and tissue accretion are similar to the human, or at least, the differences are understood. Unfortunately, lutein cannot be detected in the brain or retina of mice or pigs at normal dietary intakes (unpublished data). As noted above, recent work by Li *et al*. [13] revealed species-dependent differences in the affinity of the carotenoid cleavage enzyme, BCO2, for lutein which may explain the high lutein accumulation in primate eyes and brains. We believe the animal model of choice for investigation into mechanisms of action in brain and retina should possess a macula, and therefore, the intricate metabolic adaptations that support appropriate transport and tissue uptake and maintenance of lutein. In this regard, work previously reported by members of this team has led us to the rhesus macaque—an animal that satisfies the criteria discussed above and has been well studied with respect to lutein nutrition.

Although several studies have now reported associations between lutein intake or tissue levels and cognitive function, the underlying mechanisms are not currently understood. However, it is known that lutein is the predominant carotenoid in older adult human brain, infant human brain and macaque brain. Lutein has been found in every brain region tested in both species, can only be acquired through the diet and correlates with pre-mortem cognitive function in elderly humans. Reported pmol/g tissue concentration ranges are 100 times lower than those reported for α-tocopherol [1]. However, possible neuroprotective effects of lutein may involve its role as an anti-oxidant and, importantly, the potential for higher local concentrations have not been addressed by the current literature. Traditional analytical methods do not provide insight into the distribution of lutein within various cellular membrane pools or its relationship to the complexities of brain tissue morphological organization.

Toward overcoming these limitations, our team has concluded that there are four main research approaches that can advance basic knowledge about the role of lutein in brain development and cognitive function. The first is to determine the subcellular distribution of lutein in membrane fractions. While this approach does not preserve cellular identity, lutein accumulation in membrane fractions isolated from specific brain regions may provide clues to its functions. The second approach is to use mass spectrometry imaging which maintains the three-dimensional organization of brain slices and has the potential to detect low levels of multiple compounds. Thirdly, feeding monkeys ^13^C-lutein will potentiate the ability of these methodologies to detect low concentrations and to distinguish exogenous lutein and its isomers from the endogenous compound. The fourth approach is to examine the mechanism of delivery of dietary lutein to brain in primates. We plan to extend the current literature through application of novel, sensitive imaging combined with measures of brain function. Results from these four approaches will be used to inform nonhuman primate and human cognitive function intervention trials. For example, cognitive tasks that depend on particular brain structures could be applied in future clinical trials based on knowledge gained from this work. Specific findings from the current undertaking could also lead to the application of specific brain imaging testing in infants and adults with the goal of demonstrating improved brain development or function.

## Abbreviations

8-oxodG: 8-oxo-2-deoxyguanosine; AMD: age-related macular degeneration; BCO2: β,β-carotene-9′,10′-dioxygenase; DHA: docosahexaenoic acid; FA: fatty acid; GC: gas chromatography; GCMS-NCI-SIM: gas chromatography mass spectrometry-negative chemical ionization-selected ion monitoring; HPLC: high performance liquid chromatography; LC: liquid chromatography; LDI-MSI: laser desorption ionization-mass spectrometry imaging; MALDI-MSI: matrix-assisted laser desorption/ionization-mass spectrometry imaging; MS: mass spectrometry; NP: neuroprostanes; PM: plasma membranes; ROS: reactive oxygen species; RPE: retinal pigment epithelium.
